# 

**Published:** 1913-11-01

**Authors:** 


### Gifts and Bequests.

Under the will of the late Mrs. Jane Barber the Leicester Royal Infirmary receives £100 and the Children's Hospital £50.

Miss Alice Joanna Sarah Dorothea Scurfield, of Durham, has left £1,000 each to Ruston Hospital, Northallerton, and the Gloucester Children's Hospital.

A legacy of £200, less estate and succession duties, has been received by the Metropolitan Hospital Saturday Fund from the Executors of the late Mr. Thomas Horsman.

The Chelsea Hospital for Women has received £500 from the Trustees of the Zunz Bequest. This donation is additional to the Trustees' promise of £10,000 towards the Rebuilding Fund.

Lord Donoughmore, treasurer of the London Homœopathic Hospital, Great Ormond Street, W.C., has received £1,000 from Mrs. Jane Paterson Williams (*née* Chalmers), per Dr. George Burford, to endow the " Andrew Crichton Chalmers Bed " for a female patient in one of the paying rooms of the hospital. This is to enable poor ladies of the professional and governess class to be admitted to a private room in the hospital at a reduced weekly charge. This is the second bed endowed in these paying rooms.

